# The “*Journal of Functional Morphology and Kinesiology*” Journal Club Series: Highlights on Recent Papers in Overtraining and Exercise Addiction

**DOI:** 10.3390/jfmk4040068

**Published:** 2019-09-30

**Authors:** Antonino Bianco, Silvia Ravalli, Grazia Maugeri, Velia D’Agata, Michele Vecchio, Agata Grazia D’Amico, Vito Pavone, Ludovico Lucenti, Alessandra Amato, Ambra Gentile, Valerio Giustino, Kaltrina Feka, Ewan Thomas, Giuseppe Musumeci

**Affiliations:** 1 Department of Psychology, Educational Science and Human Movement, University of Palermo, Via Giovanni Pascoli 6, 90144 Palermo, Italy; antonino.bianco@unipa.it (A.B.); amale94@hotmail.it (A.A.); ambra.gentile01@unipa.it (A.G.); valerio.giustino@unipa.it (V.G.); kaltrina.feka92@gmail.com (K.F.); ewan.thomas@unipa.it (E.T.); 2 Department of Biomedical and Biotechnological Sciences, Human Anatomy and Histology Section, School of Medicine, University of Catania, Via S. Sofia 87, 95123 Catania, Italy; silviaravalli@gmail.com (S.R.); grazia.maugeri@libero.it (G.M.); vdagata@unict.it (V.D.); 3 Department of Biomedical and Biotechnological Sciences, University of Catania–U.O. Rehabilitation A.O.U. “Policlinico-Vittorio Emanuele”, 95123 Catania, Italy; michele.vecchio@unict.it; 4 San Raffaele Open University of Rome, Department of Human Science and Promotion of quality of Life, 00148 Rome, Italy; agata.damico@unisanraffaele.gov.it; 5 Department of General Surgery and Medical Surgical Specialties, Section of Orthopedics and Traumatology, A.O.U. Policlinico-Vittorio Emanuele, University of Catania, Via Santa Sofia 78, 95123 Catania, Italy; vitopavone@hotmail.com (V.P.); ludovico.lucenti@gmail.com (L.L.); 6 Research Center on Motor Activities (CRAM), University of Catania, 95123 Catania, Italy

## Abstract

We are glad to introduce the seventeenth Journal Club. This edition is focused on several relevant studies published in the last years in the field of Overtraining and Exercise Addiction, chosen by our Editorial Board members and their colleagues. We hope to stimulate your curiosity in this field and to share with you the passion for the sport seen also from the scientific point of view. The Editorial Board members wish you an inspiring lecture.

## 1. Introduction

Overtraining involves progressively increasing training to a level that is inappropriate for performance management. Exercise addiction is an excessive and unhealthy dependence on an exercise regime that may lead to physical, psychological and emotional damage [1]. Sport has been normally presented as a ‘good news story’, promoting healthy competition along with a healthy lifestyle [2]. While this may be true in the majority of cases, there can be a downside to physical activity which should not be ignored. This may happen where the reasons for taking part in sport and exercise have become unhealthy, and the individual begins to show symptoms that the line has been crossed—what was once a recreation or pastime now increasingly resembles an obsession [3]. By nature, endurance athletes like to push their limits in an effort to improve. For some, that drive goes beyond simply doing their best and it becomes an addiction. This addiction can lead to many problems, both physical and mental, and can have very severe consequences. Often, it is a rigid and thin social aesthetic that creates this growing problem. Overtraining is a condition in which the physiological demand of an exercise regime outweighs the ability of the body to adjust to the demand. The consequences of overtraining are widespread, negatively affecting several physiological systems, including the neuroendocrine, immunological, cardiovascular, and musculoskeletal systems, respectively. Overtraining could also result in several negative psychological disturbances. Exercise dependence is a pathology in which a person becomes addicted to exercise, often exercising twice or more daily, while experiencing physical and psychological withdrawal symptoms is also a probable cause of overtraining. Due to his or her excessive exercising, a person diagnosed with exercise dependence is equally as vulnerable to overtraining as the committed athlete [4].

## 2. Recent Papers Regarding Overtraining and Exercise Addiction 

### 2.1. Risk of Exercise Addiction in Amateur Runners

#### Highlight by Grazia Maugeri and Velia D’Agata

Running represents one of the most convenient ways to train intensely. It is easy to perform, relatively inexpensive, time efficient and easily accessible. Regular running is associated to several benefits, including a decline in the risk of developing cardiovascular and respiratory diseases, positive mental well-being, self-esteem, a significant loss of adipose tissue and circulating inflammatory molecules, and reduction of metabolic disease risk [5,6]. However, uncontrollable excessive overtraining may lead to adverse effects, increasing susceptibility to sport injuries, chronic musculoskeletal pain, alteration of human immune system or social-occupational dysfunction [7]. This new behavioral addiction having an obsessive-compulsive dimension as well as rewarding aspects, is known as “exercise addiction” (EA). In a recent and interesting study by Lukács et al. [8], EA was evaluated in amateur runners. For this aim, a total of 257 runners (48.9% females, Male = 40.49, SD = 8.99 years) with at least 2 years running activity, participated in an anonymous questionnaire survey. The prevalence of EA in amateur runners was assessed by the well-validated Exercise Dependence Scale (EDS). Moreover, the risk of EA was estimated based on a multinomial logistic regression including five variables: Duration of weekly training session; childhood sport activity; educational level; anxiety and loneliness. The results showed that prevalence of risk of EA was 8.6%, whereas 53.6% of respondents were characterized non-dependent symptomatic and 37.8% non-dependent asymptomatic. Interestingly, anxiety and loneliness may induce the amateur runners to increase the time or volume of sport activity. Furthermore, lower educational level represented a risk factor to EA development. Further research is needed to better understand the real reasons responsible of EA development. Nonetheless, this study gives a significant contribution to overall knowledge related to this behavioral addiction.

### 2.2. Sport Addiction: Overtraining and Extreme Risk

#### Highlight by Silvia Ravalli

Regular exercise has a great importance in maintaining good health, indeed inactivity is a risk factor for different chronic diseases [9,10]. Daily exercise is a strongly suggested habit but, sometimes, people could feel to be physically and mentally addicted to it and are unable to stop even when they suffer from pain or injury [11]. The term “exercise addiction” has been proposed to indicate the condition in which physical activity evolves into compulsive behaviour, leading to unhealthy risks and potentially dangerous situations [12]. The appearance of this behaviour has been seen to be connected to excessive and intense practice, associated with some specific sport categories and linked to different social and cultural aspects. In addition, propensity for high-risk activities, obsessive passion and dedication to sports are the personality traits involved in the definition of a sport-addicted [13]. The reasons behind this phenomenon rely on the social pressure for a perfect body, on the attraction to risky and dangerous situations and on the feeling of euphoria, known as “runner’s high”, provoked by the central effects of endorphins and other endogenous opioids, described by some athletes in the same manner as drug addicts talk about substances. Genetics is also thought to be involved in the sensation-seeking trait of personality which appears to be very common in those who practice extreme sports. Links between polymorphisms of the D4 subtype of the dopamine 2 receptor with risk-taking behavior in humans have been studied in order to understand the underlying mechanisms [14]. Extreme sports are defined as activities in which accidents would most likely result in serious injury or death, such as rock climbing or BASE jumping. The endurance training, psychological tolerance, tough commitment and strong motivation, which are necessary to practice these kinds of sports, seem to favour the development of adrenaline-addicted athletes.

### 2.3. Microtraumatic Peripheral Neuropathies in Overtraining

#### Highlight by Michele Vecchio

Sporting activities, especially if carried out excessively, can cause peripheral neuropathies due to micro traumatisms as sometimes described in the literature [15]. These neuropathies, related to repetitive activities, appear to be less frequent compared to neuropathies related to direct traumas in sport [16]. In a recent article by Radic Borislav et al. [17] the authors describe subacute and chronic nerve injuries are the result of overuse injuries specifying overuse injury develops as a result of numerous factors such as constant repetition of one and the same movement, permanent muscular contraction or strain, and violent movements; thus, the overuse syndrome is referred to as a gradual injury process [18]. The mechanism of overuse injury development includes athletic training regimen, relevant equipment, and periodization. The term periodization means systematic training planning with cyclic peak performance in training and with appropriate rest [19]. Authors pay attention to acute traumatic nerve lesions and also to repeated micro traumatism lesions as for (a) long thoracic nerve injury that occurs after chronic recurring heavy shoulder strains in sports such as archery, bodybuilding, judo, karate, tennis, volleyball, wrestling and golf [20]; (b) suprascapular nerve injury that rarely is injured in acute mechanical action and it is most commonly injured by recurrent hyperabduction movements in the shoulder, especially in the athletes playing baseball, volleyball and tennis; (c) the median nerve compression that is usually the result of excessive and repetitive movements in the elbow and forearm. The median nerve is compressed by two heads of the pronator teres muscle. Pronator teres syndrome has been described in archery, baseball, weightlifting and racket sport. The median nerve can also be affected by distally and carpal tunnel syndrome (is the most common compressive syndrome of the arm. It is the result of strong pressure in the wrist and is most commonly found in cycling, archery, bodybuilding, weightlifting, wrestling, golf and tennis; (d) ulnar nerve compression on the hand (Guyon’s canal syndrome) is due to a direct compressive injury in wheelchair athletes, cyclists, cross-country skiers and snowmobilers; (e) radial nerve is most often damaged in the radial sulcus on the upper arm. In athletes, it most commonly occurs as a result of excessive and repetitive elbow extension (racket sports, tennis); (f) lateral femoral cutaneous nerve can be damaged in contact sports (football and rugby), jumping sports (gymnastics with repetitive flexion and extension in the hip), or with compression from external gear as scuba diving [21], (g) femoral nerve injury occurs during collision, often with hyperextension (gymnastics, football, dancing, long jumping, basketball, ballet dancing, bodybuilding, cross-country skiing); (h) common peroneal nerve injury in sports can develop as an acute traumatic event or as overuse injury. Due to its superficial position, the nerve is subject to blunt trauma, especially in sports such as hockey, soccer and football. A special form of the peroneal nerve injury occurs in runners [22]. The nerve can be compressed by the muscle fascia; (i) deep peroneal nerve can be injured in the area of crossing from the ankle to the foot. This can usually be seen in skaters and skiers when inappropriate sports equipment compresses the nerve in the ankle area; (l) tibial nerve distal focal neuropathy is described in athletes [23]; (m) pudendal nerve entrapment (Alcock canal syndrome) is caused by prolonged sitting on bicycle seat (cyclists) [24]. The authors concluded said that peripheral nerve injuries in sports are rare and occur as a result of acute injuries. Chronic injuries are “overuse injuries” or the result of muscle and joint damage, rarely as a result of inappropriate sports equipment. Treatment involves physical therapy, analysis of nerve injury mechanisms, and occasionally surgical treatment.

### 2.4. Overactivation of the Reward System and Deficient Inhibition in Exercise Addiction

#### Highlight by Agata Grazia D’Amico

It is well recognized that physical activity enhances psychological well-being and this beneficence is directly linked to its addiction. The concept of physical exercise addiction was firstly introduced by Sachs and Pargman (1984) [25]. The authors used this term to describe the withdrawal symptoms occurred in runners during a period of physical activity-deprivation. Over the years, many assumptions are postulate to explain the cause of exercise addiction and many others clinical cases of overtraining symptoms have been reported in different kinds of sports including body building, weight lifting and martial arts. More recently, exercise addiction is classified among behavioral addictions. Substantially, this disorder becomes progressive and athletes move from healthy to unhealthy exercise pattern. The need to conduct an overtraining is often due to psychologic problems such as anxiety, confusion and depression. The diagnosis of exercise addiction is hard owing to the lack of methodological approach and any well-defined or validated criteria. However, one criteria to be consider could be represented by incapacity to reduce or stop the physical activity. Some papers have report that exercise addiction prevalence depends on physical activity, 25% in runners and 30% in triathletes [26]. More study recently [27] have highlighted the direct relation between abnormalities in the reward and inhibition systems of exercise addicts. The reward and inhibition systems are respectively relies on limbic-striatal areas, responsible to drive impulsive and automatic behaviors and on orbitofrontal-dorsolateral cortices involved in controlled behavior. Usually, concomitant event based on reward system activation and impairment of inhibition system are responsible for the loss of control during the addiction behavior. Three groups of male participants (15 exercise addicts, 18 regular exercisers, and 16 exercise avoiders) completed the Mini International Personality Item Pool, the classic go/no-go task, and the exercise-related go/no-go task. Event-related potentials were recorded during the go/no-go tasks, and correctly, performed trials were analyzed. Results have demonstrated that over activation of the reward system and damage in inhibition system may be a crucial factor for developing exercise addiction.

### 2.5. Overuse Physeal Injuries: The Effect of Overtraining in the Young Athletes

#### Highlight by Vito Pavone and Ludovico Lucenti

Overuse injuries are largely increasing in youth athlete populations [28]. Physeal injuries are exclusive to pediatric patients, usually sustained during athletic competition or more frequently during training [29]. Indeed, this type of injury develops in response to excess stress placed on immature bony, cartilaginous and soft tissue structures [30]. A recent paper by Arnold et al. [31] reviewed 24 articles about overuse physeal injury in youth athletes, in order to assess the best available evidence regarding prevention and treatment strategies in clinical practice. The repetitive stress as a mechanism of injury in young athletes was the meeting point among all the articles examined. The study evaluated overuse physeal injuries involving both upper and lower extremities. For the lower extremities, Osgood-Schlatter disease, sever disease, and Sinding-Larsen Johansson syndrome are the 3 most common overuse physeal injuries; the first 2 syndromes account for almost 20% of all pediatric overuse injuries reported in the literature [32]. They typically occur when excess stress is placed across areas with major tendon insertions. Gymnast wrist, Little League shoulder, and Little League elbow are 3 upper physeal injuries that are highly prevalent and described frequently in literature. They are due to excess compression or traction forces placed across a joint during sport [33]. Periods of rest, correction of physical training errors, enhance cardiovascular endurance, stretching programs, correction of modifiable risk factors are the main prevention strategies found in the literature. Joint immobilization and surgical intervention can be applied only in severe cases. Risk factors have a key role: nonmodifiable risk factors for overuse injuries include: Chronological age, body size, and history of previous injury; modifiable risk factors (low flexibility, excessive strength, inadequate rest, balance deficits, overtraining and inappropriate coaching styles) need to be kept to a minimum. Extended period of active rest, non-symptomatic activities, physical therapy and then progressive strength training programs and rehabilitation can permit a quick and safe return to sport. The main limitation of the study is due to the difficulty to assess the precise time of rest and the age- and injury-specific time to return-to-sport. In fact, rehabilitation programs are often vague and lacked specific return-to-sport criteria. Further and more specific studies, considering each type of sports individually, need to be done in the future.

### 2.6. Exercise Addiction and Musculoskeletal Injuries

#### Highlight by Ewan Thomas and Silvia Ravalli

Regular physical exercise has been proven to act on the human being by increasing physical abilities, and both physiological and psychological functions [34,35]. When physical exercise is practiced at low intensity, this may also act as a preventive therapy for different cardiovascular, metabolic and musculoskeletal conditions [36]. With increasing training intensity and frequency, the body will need increased recovery time in order to lead adaptation and load compensation [37]. If such recovery time is not achieved and exercise frequency and intensity remain constant, a condition known as non-functional overreaching may occur. Such condition is characterized by decreased performance together with neuroendocrine and psychological symptoms [38]. However, if such condition results prolonged for a period of over two months, this may be defined as overtraining [39]. Being that regular physical activity is associated with increased β-endorphintrlease, the promotion of neurogenesis, the promotion of the remodeling of the hippocampal synaptic circuits also including the increase of release neurotrophic proteins including BDNF and GDNF, exercise can be considered a psychoactive drug, not without any side-effects [34]. Around 3% of the population and 42% of gym practitioners has been observed to manifest compulsory behaviors in regards to the practice of exercise, not being able to stop or feeling obliged to exercise, and this is referred to as exercise addiction [39,40]. Such condition may increase the risk and severity of injuries in exercise participants. Adams et al. [1] highlight that injury to soft tissues sustained from chronic overuse is the result of microtrauma in which the usual inflammatory cell infiltration is not present, and this would result in a faulty reparative process. Such process may affect bone, tendons, and ligaments, resulting in an increased probability of developing rotor cuff tendinosis, lateral or medial epicondylitis, patellar tendinosis, iliotibial band friction syndrome, plantar fasciitis or a stress fracture. Moreover, muscular adaptation to overtraining results in the loss of flexibility and range of motion which further contributes to a loss of articular function [41]. Kibler et al. suggested that during overuse, even a minor change in any biomechanical parameter may become greatly pronounced, indicating that once an injury has occurred in a system, there is a high probability that other systems may also be influenced. Exercise addiction represents a condition that would favor the onset and maintenance of musculoskeletal disorders; therefore athletes with a history of multiple overuse injuries may also present exercise addiction [42].

### 2.7. Overtraining and Exercise Addiction: What Psychology Says

#### Highlight by Ambra Gentile

The benefits of a regular exercise for people’s health are well known, both for optimal body and mental functioning. However, during the past decades, psychologists and sport scientists have recognized that physical exercise can also represent a negative aspect in the individuals’ life. It happens quite often that the athletes overload their bodies during their training, that, in the long term, may harm them, both physically and mentally. Even though in case of overtraining the individual still controls the behavior, the underlying attitude might turn it into exercise addiction, defined as a compulsive engagement in physical activity with detrimental effects for personal, social and professional life, without thinking about the harmful health consequences [39,43,44]. The main explanations of this phenomenon offered by scientific literature rely on physiological processes, as physical addiction developed towards the endorphins production during training, and psychological mechanisms, as motivation to improve body shape or weight-loss. As noticed by Marques et al. [43], even though it is not included into the Diagnostic and Statistical Manual for Mental Disorders (DSM-5), exercise addiction should be considered as behavior addiction, since the typical symptoms can be observed, as mood disturbance and tolerance, relapses, behavioral miscontrol, abstinence syndrome and excessive time dedicated to the activity [44]. Moreover, people affected by this disorder show also the presence of other addictions, such as nicotine, alcohol, or other drugs, eating disorders, sex addiction or shopping addiction [44]. Nevertheless, addictive behaviors represent a way to cope with stressful situations; thus people may intentionally engage in physical activity as a way to escape from reality, without caring of negative consequences for the body, because physical activity is traditionally considered as a healthy behavior [39]. Therefore, identifying exercise addiction can be difficult, since the borders between healthy behavior or commitment and pathology are not always observable, and the individuals might be not able to recognize the problem. 

### 2.8. Overtraining and Exercise Addiction: It Is Possible to Predict the Onset?

#### Highlight by Alessandra Amato

Overtrain syndrome (OTS) is characterized by acute fatigue feeling and reduced performance [45]. Recent EROS study (Endocrine/Metabolic Responses on Overtraining Syndrome) underline that to date, there are no reliable or precise biomarkers which help to diagnose overtraining [46,47]. However, Cadegiani (2017, 2019) showed the decrease in the testosterone/cortisol ratio, the increase in night urinary catecholamines, altered levels in maximum lactate concentration and creatine kinase compared to healthy athletes, [45,47,48] for this reason initial screening blood work can be useful to assess the body’s response to the training load. According to the findings mentioned above, the relationship between hormones and overtraining syndrome appear to be complex. Whenever hormonal dysfunction causes the performance to deteriorate we cannot define an OTS condition; on the contrary, OTS can lead to dysfunctional hormones, as a result, [45] the diagnosis of OTS can only be made after exclusion of the much more common medical conditions of athletes. However, some preventive strategies such as training periodization, RPE monitoring, volume and intensity of training regulation, adequate caloric intake, hydration, sleep, and constant psychological monitoring, can be applied [45,46,47,48,49,50]. Overtraining is often associated with another disorder: The Exercise Addiction. Morgan (1979) described it as the concept of addiction could also be applied to excessive exercise, mainly through the presence of withdrawal symptoms, harmful and different social consequences and psychological disorders [51]. The society acts as the most important reinforcement of these behaviors. The definition of diagnostics must, therefore, consider personality characteristics and environmental factors. Furthermore, there may be target populations in which we identify a higher percentage of people at risk of addiction to exercise such as endurance athletes [52]. Consequently, Exercise Addiction could be a cause of overtraining underlining the interdependence between the two concepts. However these are real pathologies and as such must be treated. In my opinion, other studies are necessary to investigate the diagnostic criteria.

### 2.9. Overtraining and Exercise Addiction: Doing More not Correlate to Better Results

#### Highlight by Kaltrina Feka

Physical activity (PA) is widely known for its benefits [53]. However, over the years studies have shown that doing more, at the same level does not correlate to better results. Indeed, it leads quite to the opposite effect [45,54]. When PA is not used accordingly to body abilities, several health problems may occur, both physical and mental [55]. A practical example when PA is not used accordingly to humans’ limitations is overtraining (OT), which is considered as an imbalanced condition between physiological performance, current functional capacity, and training demand [1]. Furthermore, based on the overload and intensity, overtraining may appear as acute or chronic condition. Moreover, as a result of long-term training or excessive exercise a syndrome known as overtraining syndrome may be generated. Overtraining syndrome may occur due to an increase in the intensity or duration (or both) of the exercise without a suitable program to their physiological capabilities. According to several publications, physical activity may harm both physiological and psychological processes [45,54]. Regardless the knowledge published across the world on PA, there are people who are addicted to exercise and will keep training no matter the causes. Although, exercise addiction is not considered yet as a mental health disorder [56] remains a critical issue. According to the literature, there are ways on diagnosing and treating people with exercise addiction, despite all these publications there is a lack of information on how widespread it is and which may be the most effective treatment for this specific population [44,51]. Furthermore, like all the other addictions, also this one requires attention from the therapist. However, the goal of therapy is not to prevent the patient from working out but helping them to diminish exercise routine rigidity [56]. When adequate intensity, duration of physical activity and recovery meets one another, tons of health problems may be avoided.

### 2.10. Primary Exercise Addiction and Overtraining in Children.

#### Highlight by Valerio Giustino

Exercise addiction is a widely known phenomenon among adult non-professional sports practitioners as well as in athletes. However, a few studies in the literature have shown the presence of exercise addiction among youth [57,58,59]. Tsai et al. have reported that exercise addiction represented a standard addictive behavior in the adolescents recruited for the study [57]. Recent research showed that exercise addiction was the second most common problem behavior in Italian students [58]. Matos et al. found that about 30 percent of young English athletes have experienced overtraining [59]. Given the growing number of primary exercise dependence in children and emerging adults, Lichtenstein et al. have developed and validated a version of the Exercise Addiction Inventory for young in order to assess the prevalence and the incidence of this addiction disorder [60]. This 6-item instrument includes a question concerning the overtraining syndrome as effect of exercise addiction [60]. Indeed, although a sedentary lifestyle is an alarming behavior problem for children and adolescents [61], overtraining risk among young athletes is high due to the increasing number of competitions and related training sessions in which they participate [62]. This condition increases the risk of overuse injuries and, furthermore, could induce youth to leave physical activity which, instead, should aim to entertain and induce them into a lifelong active lifestyle [63,64,65,66]. Although there are no guidelines, according to the American Academy of Pediatrics Council on Sports Medicine and Fitness the ideal regimen of sports activity for young athlete consisting of 5 days/week with 1–2 days/week and 3 months/year of rest in order to prevent overtraining and overuse injuries [67]. Among the recommendations to avoid the potential risk of overtraining, it is recommended to encourage young athletes in early age to participate in different sports and avoid sports specialization.

### 2.11. The Dangers of Exercise Addiction: Novel Insights of Overtraining Syndrome Discovered From the EROS Study

#### Highlight by Giuseppe Musumeci

Moving daily has positive effects on people’s physical and mental health [68]. Nutrition and regular physical activity, even if moderate, helps to improve all aspects of quality of life [69]. In contrast, poor physical activity, such as sedentary life, is implicated in the onset of some of the most common disorders and diseases, but if the physical activity, it is performed inadequately or in excesses, such as overuse or “overtraining syndrome” (OTS), can do much more harm than sedentary life [70,71,72]. Based on the current literature the pathophysiology of OTS today is already unclear. In a recent and interesting article by Cadegiani and Kater [73], the authors talk about excessive training and inadequate recovery could cause OTS, which is characterised by underperformance and fatigue. The authors of this study aimed to describe novel mechanisms and risk factors associated with OTS, and thereby facilitate its early identification and prevention, from a comprehensive joint qualitative analysis of the findings from all the four arms of the Endocrine and Metabolic Responses on Overtraining Syndrome (EROS) study. They compared the types and proportions of behavioural patterns of 67 evaluated parameters of OTS from 51 participants-athletes with OTS (OTS, *n* = 14), healthy athletes (*n* = 25) and healthy non-physically active controls (*n* = 12). A total of 44 (65.7%) markers exhibited significant differences between the three groups: 32 (72.7%) showed a loss of the conditioning effect of exercise (‘deconditioning’), 7 (15.9%) showed changes exclusive to OTS, 3 (6.8%) maintained the exercise-induced conditioning effects and 2 (4.5%) revealed an exacerbation of the adaptive changes to exercises. OTS was demonstrated to be a clinical state, which is induced by a distinctive combination of chronic stressors, leading to several synergistic combinations of metabolic, hormonal, inflammatory, immunological, neurological, cardiovascular and psychological dysfunctions that cause a dysfunctional “hypometabolic” and “hyporesponsive” state. These data suggest that OTS is not a simple consequence of “overtraining”, but resulted from a combination of different risk factors and harmful clinical behaviours, among which some were unprecedentedly recognised, including insufficient caloric, protein or carbohydrate intake, bad sleep quality and excessive concurrent cognitive effort. The most remarkable aspect of OTS is, therefore, not “overtraining”, but a blend of conditioning shortfalls, or ‘unexpected and paradoxical deconditioning’ [48,74]. The loss of multiple conditioning processes naturally leads to a loss in the physical conditioning, clinically observed as reduced performance. The new findings on OTS allowed us to propose that a comprehensive analysis of the athlete, including the evaluation for risk factors, early clinical signs, and relatively altered biochemistry and hormones, should be the most effective approach for the prevention of imminent OTS, prior to the occurrence of decreased performance. In conclusion their findings suggest that OTS is likely triggered by multiple factors, not restricted to excessive training, resulted from a chronic energy deprivation, leading to multiple losses in the conditioning processes typically observed in healthy athletes, as a combination of “paradoxical deconditioning” processes, which explains the gradual and marked loss of physical conditioning found in OTS. The authors concluded that “paradoxical deconditioning syndrome of the athlete” is a more appropriate and descriptive name for OTS than the previous misnomer, which is focused only on excessive training [73].
